# A34 EVALUATION OF GASTRIC ENDOSCOPIC SUBMUCOSAL DISSECTION (ESD) EFFICACY IN A SINGLE CANADIAN CENTER

**DOI:** 10.1093/jcag/gwae059.034

**Published:** 2025-02-10

**Authors:** T Maniere, D Kaufman

**Affiliations:** Gastroenterology, Hotel-Dieu de Sherbrooke, Sherbrooke, QC, Canada; Gastroenterology, Hotel-Dieu de Sherbrooke, Sherbrooke, QC, Canada

## Abstract

**Background:**

Endoscopic Submucosal Dissection (ESD), developed in Japan, is a minimally invasive method for resecting large superficial gastric lesions. It allows high en-bloc resection rates, reducing local recurrence and enabling complete histological assessment, overcoming the limitations of Endoscopic Mucosal Resection (EMR). Although well-studied in Asia, ESD’s evaluation in North America remains limited due to lower gastric cancer incidence.

**Aims:**

The aim of our study is to evaluate gastric ESD’s effectiveness and assess peri-procedural complications

**Methods:**

This retrospective cohort study included 75 patients who underwent ESD for gastric adenocarcinoma, neuroendocrine tumors, high-grade and low-grade dysplasia at Charles Le Moyne Hospital between 2017 and 2024. Data collected included patient demographics, procedural details, and pathology outcomes. Primary outcomes were en-bloc and R0 resection rates, while secondary outcomes included complications like perforation and hemorrhage. Five patients were excluded from the R0 and curative resection analysis due to non-evaluable pathology:piece meal resection (1), unconclusive margins (2), failure of resection(2).

**Results:**

A total of 75 patients were included, with a mean age of 73 years (), and 64% were men. Polyp characteristics based on Paris classification were: 0-IIa+c (21%), 0-IIa (20%), 0-Is (17%), 0-IIB (9%), 0-Ip (4%), 0-Is+IIa (4%), 0-IIc (4%), 0-Isp (3%), 0-IIa+b (3%), and 0-IIb+c (1%). Lesions were located in antrum (32), body (25) GE junction (14), angulus (5) and gastro-jejunum anastomosis (1). One patient had two polyps resected concurrently (0-Ip + 0-Is). Paris class identification was missing in 13% of cases. The mean maximum endoscopic size visual estimation was 24 mm (5-90), while the mean maximum pathology specimen size was 40 mm (8-75). Pathology results showed: 25% invasive adenocarcinoma, 18% intra-mucosal adenocarcinoma, 18% high-grade dysplasia, 20% low-grade dysplasia, 11% hyperplastic polyps, 3% neuroendocrine tumors (grades 1-2), and 2% had no pathology due to complications. The mean procedural time was 117 minutes (40-412). Perforations occurred in 17% (13/76) of patients, with successful endoscopic closure in 92% (12/13) of cases. One patient required surgical repair. En-bloc and R0 resection rates were 80% and 73%, respectively, with curative resections in 67% of cases.

**Conclusions:**

ESD demonstrated high en-bloc and R0 resection rates, confirming its effectiveness for managing gastric lesions. These results are consistent with North American studies. Though complications like perforation occurred, most were managed endoscopically. This provides preliminary evidence for ESD as a safe and effective option for gastric lesion resection in Canada.

**Funding Agencies:**

None

